# Editor’sComment

**DOI:** 10.1590/S1677-5538.IBJU.2016.01.02

**Published:** 2016

**Authors:** Sidney Glina

In the current year, the Brazilian Society of Urology celebrates its 90th year of continuous activity and the official scientific journal of the society “International Brazilian Journal of Urology” will present several important modifications throughout this festive year.

The electronic Int Braz J Urol page (www.brazjurol.com.br) was updated and became more interactive and friendly, facilitating the access to all information. From now on, it is possible to directly access all articles published from the first publication of the Brazilian Journal of Urology (the former name of our magazine), providing instant consultation. Visit the new webpage in www.intbrazjurol.com.br

Finally, Int Braz J Urol will become part of Pubmed Central, the free access to the digital collection of the National Center for Biotechnology Information (NCBI). With this achievement, our Journal will be more widespread in the international scientific community.

Read Int Braz J Urol, publish at the Int Braz J Urol, quote the good papers of Int Braz J Urol!

*Sidney Glina, MD, PhD* Editor-In-Chief Internacional Braz J Urol

